# Seasonal differences in jump performance in the Svalbard rock ptarmigan (*Lagopus muta hyperborea*)

**DOI:** 10.1242/bio.20147930

**Published:** 2014-03-21

**Authors:** John J. Lees, Lars P. Folkow, Jonathan R. Codd, Robert L. Nudds

**Affiliations:** 1 Faculty of Life Sciences, University of Manchester, Manchester M13 9PT, UK; 2 Department of Arctic and Marine Biology, University of Tromsø, Tromsø 9037, Norway

**Keywords:** Arctic, Biomechanics, Birds, Body composition, Fat, Locomotion, Predation, Take-off

## Abstract

Fat storage is essential to the survival of many bird species, providing energy reserves, but can have an effect on locomotor performance with an associated potential increase in predation risk. In particular, the ability to initiate flight through jumping is critical to predator avoidance and may be influenced by changes in body mass (*M*_b_). Here we investigate seasonal differences in the jump take-off performance of high Arctic Svalbard rock ptarmigan (*Lagopus muta hyperborea*) resulting from around a 50% increase in *M*_b_ during winter as a result of fat deposition. Using force-plate data and videography, we reveal that, in the absence of alterations to take-off angle, winter Svalbard rock ptarmigan are unable to increase hind-limb power output during jumping to compensate for their increased *M*_b_. As a result, peak take-off velocity is reduced by 42% and jump duration is also extended during winter. The consequences of reduced jumping performance upon Svalbard ptarmigan during winter may be relatively small given their low risk of predation during this season. It may be, however, that the observed reduction in jumping performance when fat may contribute to the sub-maximal pattern of fat acquisition observed in other bird species.

## INTRODUCTION

The seasonal and diurnal acquisition and maintenance of fat reserves is important to many avian species. Birds commonly rely on fat for insulation and as an energy source to be utilized during periods of food scarcity, low temperature or during migration (King, 1972). There are, however, associated costs that increase with the level of stored fat. As a result, birds seldom maintain maximal fat reserves and stores are instead regulated through a cost versus benefit trade-off (Gosler et al., 1995; Witter and Cuthill, 1993). The primary benefit is a lowered risk of starvation, whereas costs are related to the increases in energy expenditure and predator susceptibility as a result of elevated body mass (*M*_b_) (Brodin, 2001). The relative importance of these costs will vary with factors such as food availability and the degree of predation, providing a direct link between the environment and the physiological or behavioural strategies of birds (Lima, 1986). Energy expenditure may increase as a result of the mass-dependent cost of locomotion (both terrestrial and aerial), thereby reducing net gain during foraging (Taylor et al., 1980; Witter and Cuthill, 1993). An increased need to forage in order to meet these higher energy demands may also increase the risk of predation. In combination with impaired escape performance as a result of the increased loading, these factors may have significant consequences for mortality rates (Kullberg et al., 1996; Metcalfe and Ure, 1995; Veasey et al., 1998). Understanding the various costs associated with fat storage is therefore fundamental to multiple aspects of avian behaviour and physiology, including energy storage strategies, migratory and seasonal behaviour, population-size regulation and parent–offspring relations.

Jumping is an important part of the locomotor repertoire for birds, as they rely on it to become airborne. Furthermore, this behaviour forms the primary escape response of most avian species from aerial and terrestrial predators, particularly when the flight is initiated from the ground. The maintenance of effective jumping performance is therefore critical to survival (Veasey et al., 1998). For example, redshanks (*Tringa totanus*) suffer 83% higher mortality when they are unable to get airborne before attack from sparrowhawks (*Accipiter nisus*) (Cresswell, 1993). However, despite its importance, jumping has received less attention than terrestrial locomotion or flight but is likely to be impeded through the acquisition of fat reserves. Jump take-off in birds involves the rapid contraction of the hind-limb muscles in order to generate the high powers required to accelerate the body to the velocities needed for the commencement of flight (Provini et al., 2012). It is the high rate of work that must be done that ultimately constrains jumping performance, as power requirements cannot exceed the power output capacity of skeletal muscle (unless a mechanism for power amplification is employed). This reduced capacity is particularly problematic in smaller species whose shorter limbs constrain the distance over which the animal can accelerate (Bennet-Clark, 1977). Additionally, in accordance with Newton's second law, during fattening, jumping acceleration will be impaired if individuals cannot meet the increased power demands of elevated *M*_b_. Indeed, a number of observations indicate that small passerines have difficulty taking off when body mass is elevated (Blem, 1975; Fry et al., 1970; Metcalfe and Ure, 1995; Veasey et al., 1998; Witter and Cuthill, 1993). Empirical studies in passerine species using a variety of methodologies found differing effects of elevated *M*_b_ upon jump performance. One approach used is to artificially elevate the masses of birds using externally attached loads and measure performance variables such as velocity and take-off angle from video data. In European starlings (*Sturnus vulgaris*) adding loads of between 7 and 14% of *M*_b_ resulted in a reduced take-off angle, allowing maintenance of velocity (although flight manoeuvrability decreased) (Witter et al., 1994). Conversely, zebra finches (*Taeniopygia guttata*) maintained take-off angle, but kept energy expenditure at pre-loaded levels by reducing velocity, when carrying loads of 27% of their *M*_b_ (Nudds and Bryant, 2002). Some studies utilizing videography to assess take-off performance have included species that are ‘naturally’ loaded due to the acquisition of fat reserves or eggs. The findings of these studies are mixed but appear primarily influenced by the amount of fat gained. For example, blackcaps (*Sylvia atricapilla*) decreased take-off angle and velocity in response to 60% increases in *M*_b_ (Kullberg et al., 1996), whereas willow tits (*Poecile montanus*), great tits (*Parus major*), yellowhammers (*Emberiza citrinella*) and greenfinches (*Carduelis chloris*) with 7–8% increases in *M*_b_ were unaffected (Kullberg, 1998; Kullberg et al., 1998; van der Veen and Lindström, 2000). European robins (*Erithacus rubecula*) with intermediate *M*_b_ increases (27%) were able to reduce take-off angle in order to maintain velocity (Lind et al., 1999), similar to the findings in artificially (Witter et al., 1994) but not naturally loaded (Lee et al., 1996) starlings. Therefore, although *M*_b_ clearly has a significant influence upon take-off performance, artificial loading experiments can be hard to interpret and do not necessarily mimic the situation in wild birds. Similarly, comparison of results may be hampered by methodological inconsistencies in the methods used to elicit a take-off response (escape response versus normal take-off), coupled with differences in the calculation of performance indicators.

To date, studies of the effects of mass gain have generally focused on passerines taking off from perches. Rapid take-off, however, is equally important to ground-dwelling species, such as phasianids, which rely on anaerobic, burst take-off to escape terrestrial predators (Henry et al., 2005; Tobalske and Dial, 2000). Indeed guinea fowl (*Numida meleagris*) are capable of exceptional take-off performance, with the legs producing peak vertical forces of 5.3 times body weight during jumps (Henry et al., 2005). Additionally, their legs are capable of storing elastic energy early in the jump, which is later released, allowing power outputs of more than twice those predicted from their hind-limb muscle-mass alone (Henry et al., 2005). Similarly, the hind-limbs of quail (*Coturnix coturnix*) and European starlings produce forces of 7.8 and 4 times body weight, respectively, yielding 80–90% of the velocity required for ground take-off (Earls, 2000). Despite the large number of terrestrial birds that undergo seasonal fattening and the importance of jump performance, the influence of naturally elevated *M*_b_ upon hind-limb driven ground take-off is unknown.

The Svalbard rock ptarmigan (*Lagopus muta hyperborea* Sundevall, 1845) is a ground-dwelling phasianid bird resident on the Arctic archipelago of Svalbard. It is non-migratory and individuals undergo marked seasonal variations in *M*_b_. During summer, birds weigh around 500 g. Prior to the onset of winter, however, *M*_b_ rapidly increases, and may double by mid November (Stokkan et al., 1986). These annual changes in *M*_b_ are attributable to seasonal fluctuations in fat stores (comprising around 32% of *M*_b_ during winter), serving as an energy source during times of limited food availability (Mortensen et al., 1983). These stores, however, cannot alone see birds through the winter, meaning they must maintain foraging activity. Previous research into the energetic consequences of the Svalbard ptarmigan's seasonal fat gain demonstrated remarkable adaptations towards efficiency of load carriage whilst walking in the winter (Lees et al., 2010). However, despite incurring no metabolic cost, the additional winter fat load significantly reduces the running speed that birds are capable of (Lees et al., 2010). How this fat load affects other aspects of locomotor performance involving the hindlimbs is yet to be determined in this species. It is possible that they also have mechanisms to ameliorate the effects of additional mass during the take-off jump. Performance variables such as force and power production, and ultimately jump velocity generated by the hindlimbs alone are important as they initiate take-off. Hence, the hindlimbs provide the baseline upon which the wings later build and their performance is likely decisive in escaping a ground predator. Understanding how such variables are influenced by the seasonal acquisition of large fat stores is not only relevant to this Arctic bird but also to other ground-dwelling birds exhibiting fluctuations in *M*_b_.

Here the impacts of natural seasonal changes in *M*_b_ upon the jump take-off performance of the Svalbard rock ptarmigan were investigated using ground reaction force measurements. Given the reduced running speed capabilities of the Svalbard rock ptarmigan in winter (Lees et al., 2010), we hypothesized that during winter, birds will also demonstrate reduced jump performance due to an inability to meet the increased power demands imposed by an elevated *M*_b_.

## RESULTS

**Fig. 1.**
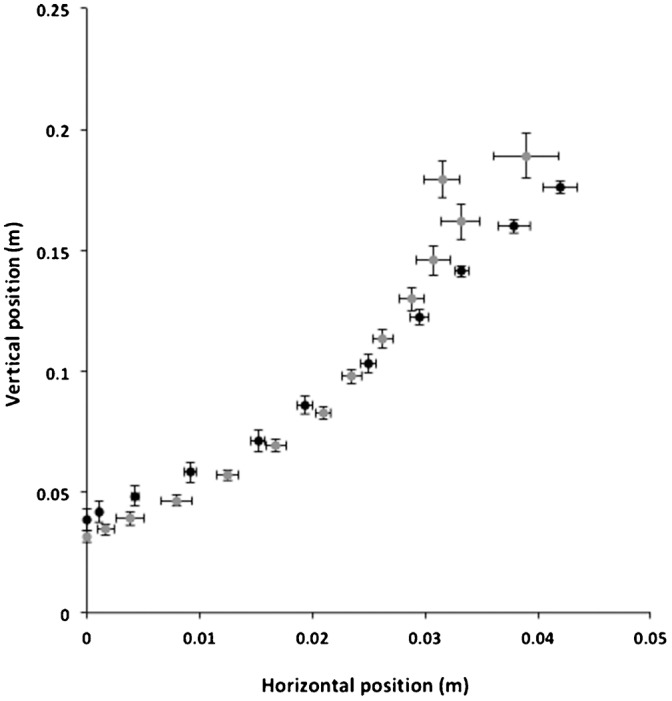
Sternal position during jumping. Comparison of the horizontal and vertical positions of the sternum of Svalbard ptarmigan throughout the duration of the jump during summer (black circles, *n* = 5, *M*_b_ = 551.5±9.4 g) and winter (grey circles, *n* = 4, *M*_b_ = 806.6±26.3 g). All data points represent the mean ± SE.

**Fig. 2.**
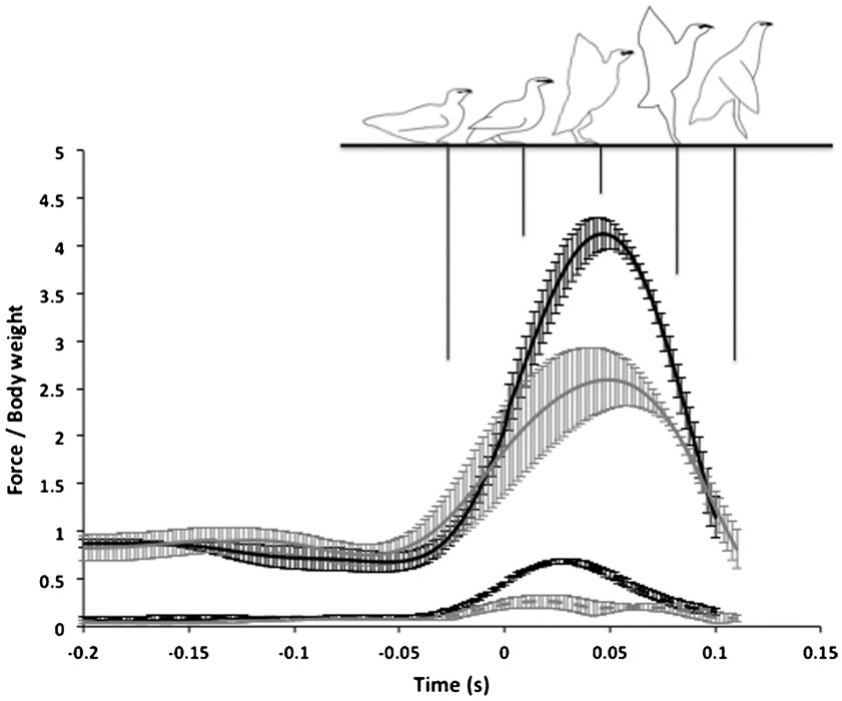
Mass-specific jumping forces. Mean vertical (solid) and horizontal (dashes) force/body weight ratio during the different stages of the jump in summer (black, *n* = 5, *M*_b_ = 551.5±9.4 g) and winter (grey, *n* = 4, *M*_b_ = 806.6±26.3 g).

During both seasons the mechanics of the jumps were the same, with the birds briefly adopting a crouched position immediately before the jump itself. Although the duration of this crouch appeared to be higher and more variable during summer (160±50 ms) than winter (100±9 ms), a clear difference was not detectable (*t* = 1.293, *df* = 8, *P* = 0.232). After the initial crouch the sternum ascended (marking the start of the jump), moving forwards and upwards as the legs extended (Fig. 1). The wings unfolded around 40–60 ms into the jump and following a flexed wing upstroke, the downstroke started at a similar time point (*t* = 0.324, *df* = 8, *P* = 0.754) during summer (110±1 ms) and during winter (110±4 ms) (Fig. 2). Toe-off (the point at which any contact with the ground ended) was sooner (*t* = 6.43, *df* = 8, *P*<0.001) during summer (102±2 ms) than winter (125±3 ms). Therefore, the downstroke occurred after toe-off during summer and prior to toe-off during the winter. The horizontal and vertical paths of the sternum were similar throughout the duration of the jump during both seasons, validating the method for maintaining a similar take-off angle (Fig. 1). Although take-off body angle appeared lower in summer (−4.6±4.9°) than winter (−12±6.69°), the large amount of variability between individuals meant that this was not supported statistically (*t* = 0.923, *df* = 8, *P* = 0.38).

**Fig. 3.**
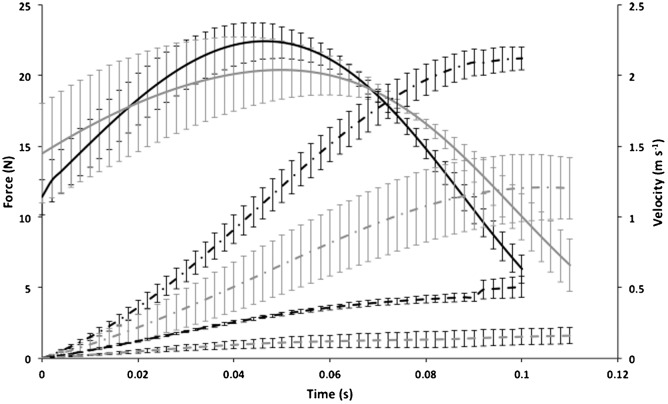
Mean force and velocity during jumping. Mean vertical forces (N, solid lines ± SE) and horizontal (dashes), and vertical (dots and dashes) velocities (m s^−1^ ± SE) during summer (black, *n* = 5, *M*_b_ = 551.5±9.4 g) and winter (grey, *n* = 4, *M*_b_ = 806.6±26.3 g).

Peak vertical forces in summer and winter were 22.91±1.12 N and 21.53±1.91 N, respectively (Fig. 3; Table 1). These values were equivalent to 4.21±0.15 and 2.73±0.28 times body weight, during summer and winter, respectively (Fig. 2; Table 1), indicating a clear seasonal difference (*t* = 4.92, *df* = 7, *P*<0.01). The timing of peak vertical force generation did not differ between seasons (*t* = 0.135, *df* = 7, *P* = 0.9) occurring at 48±3 ms and 49.1±10 ms after the start of the jump during summer and winter, respectively (Figs 2 and 3). Peak horizontal forces differed (*t* = 2.96, *df* = 7, *P*<0.05) between summer (3.99±0.12 N or 0.74±0.029 times body weight) and winter (2.75±0.45 N or 0.35±0.058 times body weight), but occurred at the same time (30 ms) into the jump (*t*-test; *t* = 0.144, *df* = 7, *P* = 0.889) (Fig. 2; Table 1). The overall result of similar force magnitude in both summer and winter was a reduction of both peak vertical (*t* = 4.212, *df* = 7, *P*<0.01; summer = 2.13±0.076 m s^−1^, winter = 1.23±0.22 m s^−1^) and peak horizontal velocities (*t* = 4.703, *df* = 7, *P*<0.01; summer = 0.44±0.032 m s^−1^, winter = 0.18±0.05 m s^−1^) in winter compared to in summer (Fig. 3). Peak vertical power was consequently reduced (*t* = 2.802, *df* = 7, *P*<0.05) in winter birds and was 34.49±2.41 W and 20.59±4.71 W in summer and winter, respectively (Table 1). Peak horizontal power also differed (*t* = 3.25, *df* = 7, *P*<0.05) between summer (1.01±0.15 W) and winter (0.35±0.13 W) (Table 1). As a consequence, average total power was higher (*t* = 2.914, *df* = 7, *P*<0.05) in summer birds, as was average body mass-specific total power (*t* = 5.236, *df* = 7, *P*<0.01) (Table 1).

**Table 1.**
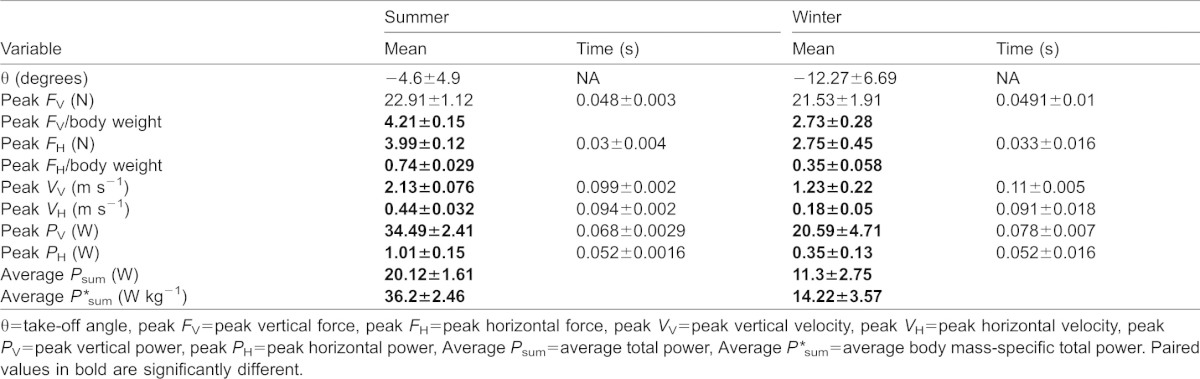
Mean values and time of occurrence of the measured jump performance variables during summer and winter

## DISCUSSION

The results of this study support the hypothesis that an increased *M*_b_ compromises jump performance in Svalbard rock ptarmigan and potentially other species as well. Increasing power requirements associated with the seasonal acquisition of body fat clearly places constraints upon the ability to jump and therefore, to become airborne from the ground. In summer, jumping performance was consistent with that found in other bird species. The 200 ms duration of the pre jump crouch lies between previously reported values in guinea fowl, which utilize a squat jump (400 ms crouch (Henry et al., 2005)), and starlings, which utilize a countermovement jump (106 ms (Earls, 2000)). The observation of vertical ground reaction forces less than body weight 100 ms prior to the start of the jump is similar to that measured in starlings and suggests ptarmigan use a form of countermovement jump. This reduction in force may serve to enhance power output as elastic elements are stretched prior to and during the extension phase of the jump, enabling the storage of elastic energy that can be recovered later in the jump (Aerts, 1998; Henry et al., 2005). Countermovement jumps are not just used by birds, but are also used by humans (Alexander, 1995; James et al., 2007) and bats initiating jumps using their forelimbs (Gardiner and Nudds, 2011). Following the pre-jump crouch, the vertical force signature of the jump itself is symmetrical, similar to that of the quail and is indicative of a rapid take-off (Earls, 2000). Unlike that of guinea fowl and during the descending phase of the force trace in vampire bats (*Desmodus rotundus*) there is no characteristic ‘shoulder’ present in the trace that is thought to be associated with sequential activation of the hind-limb muscles early in the jump (Henry et al., 2005; Schutt et al., 1997). The peak vertical force of four times body weight half way through the jump is similar to other values found in ground-dwelling birds proficient at take-off such as the Japanese quail, *Coturnix coturnix japonica* (3.9 times body weight) (Clark and Alexander, 1975) and guinea fowl (5.3 times body weight) (Henry et al., 2005), but lower than those in common quail, in which peak force is 7.8 times body weight (Earls, 2000). The resultant vertical velocity and power were sufficient to allow the summer ptarmigan to successfully make the transition to flapping flight. Indeed the peak vertical velocity value of 2 m s^−1^ exceeds that of other species during both perch (Provini et al., 2012) and ground (Earls, 2000) take-off. Summer birds clearly demonstrate the capacity for effective predator escape in the wild. During winter, however, absolute vertical ground reaction forces were not increased to meet the demands imposed by the greater *M*_b_. Although the duration of the jump was increased by almost 25% (not evident from Fig. 2 as only mean data available for all individuals are plotted) and the ascending limb of the vertical force signature was less steep, a reduction in both peak vertical velocity and power in winter to around half that recorded in summer ptarmigan was evident (Table 1). This halving of performance variables is not proportional to the percentage mass increase, which was only around 50%. Although forces exceeding body weight may be sufficient to initiate flight, this reduction in jump performance would presumably have significant consequences for the ability of winter ptarmigan to escape predation in comparison to summer birds, particularly as the additional mass is also likely to compromise flight performance. The only species to exhibit such low leg forces in comparison to body weight so far are pigeons (*Columba livia*) and rufous hummingbirds (*Selasphorus rufus*), which produce around 1.6 times body weight but likely compensate for this by high levels of wing-driven lift generation (Heppner and Anderson, 1985; Tobalske et al., 2004). The reduced take-off performance of winter Svalbard ptarmigan is consistent with data from the only comparable avian study in terms of the percentage of mass increase, in which blackcaps (60% elevation in *M*_b_) showed a 32% lower angle of ascent and 17% lower take-off velocity than lean individuals (Kullberg et al., 1996). Although the magnitude of this reduction is less than our value of 42%, despite blackcaps being relatively heavier, the study of Kullberg et al. included wing-driven increases in velocity, used a simulated predator stimulus and take-off angle was not restricted (Kullberg et al., 1996). Perhaps unsurprisingly, given the small increases in *M*_b_ (7–8%), other studies have found even smaller effects of elevated *M*_b_ upon take-off performance in birds faced with a simulated predator (Kullberg, 1998; Kullberg et al., 1998; Lind et al., 1999; van der Veen and Lindström, 2000). It is reasonable to assume a startling stimulus would initiate maximal jump performance. The motivation to jump in winter ptarmigan, however, may be reduced, perhaps to avoid any damaging effects of high-powered movements whilst heavy. Regardless of this, even if motivation does add to the reduced jumping performance of Svalbard ptarmigan, this does not change our conclusions that overall winter birds are compromised in their jumping ability when tested under identical conditions to those of summer birds. There was no evidence to suggest an habituation effect, with only 1 bird (winter) producing its best jump performance on its first analysed jump. As opposed to performing sub-maximally during winter, ptarmigan may alternatively be performing maximally, but operating at their physiological limits during this time. Winter Svalbard ptarmigan are unable to aerial run, an energy efficient mode of locomotion in summer birds (Nudds et al., 2011), despite a reduced metabolic cost of terrestrial locomotion at all speeds (Lees et al., 2010). In the absence of an apparent metabolic limit upon speed, the decreased maximum running speed in winter birds may be because they are unable to withstand the larger hind-limb forces associated with high-speed gaits (Biewener, 1998; Biewener, 2003), with muscle and tendon springs in the legs becoming overloaded (Konow et al., 2012). The hindlimbs of winter birds may similarly be overloaded during jumping and unable to meet the increased power requirements imposed by their mass.

Robins, blackcaps and starlings decrease their take-off angle when loaded (Kullberg et al., 1996; Lee et al., 1996; Lind et al., 1999; Witter et al., 1994). In two of these cases, take-off velocity was maintained (Lind et al., 1999; Witter et al., 1994). Zebra finches with 27% loads, on the other hand, demonstrated a reduced take-off velocity with no change to take-off angle (Nudds and Bryant, 2002). Nonetheless, a reduction in take-off angle may provide a mechanism by which birds can maintain take-off velocity when carrying additional *M*_b_. The relative importance of velocity and angle, however, may vary among species, depending upon the nature of the predation risk and the physical environment (Lima, 1993). For example, birds avoiding terrestrial predators presumably benefit from the maintenance of a more vertical take-off (Witter and Cuthill, 1993). Our experimental design ensured a vertical take-off and when artificially loaded, humans performing vertical jumps showed similar reductions in take-off velocity to those in the ptarmigan (Cormie et al., 2008; Davies and Young, 1984).

Birds are known to actively regulate levels of fat in order to minimize the consequences of body loading such as foraging/locomotor costs and predatory costs (Witter and Cuthill, 1993; Gosler et al., 1995). A quantification of these costs is therefore fundamental to understanding the patterns of mass change in wild birds. In birds carrying significant fat reserves such as the Svalbard ptarmigan, we might expect to observe adaptations towards ameliorating the risk of predation. Winter ptarmigan are able to carry their extensive lipid reserves at no extra metabolic cost during terrestrial locomotion and foraging (Lees et al., 2010). Yet they are severely restricted in terms of running and jumping performance. Predation, however, is generally lower on birds at high latitudes (McKinnon et al., 2010) and the only predator posing a risk to the ptarmigan, the Arctic fox (*Vulpes lagopus*), is an opportunistic scavenger, that itself relies heavily on body fat and food caches (consisting primarily of Svalbard reindeer, *Rangifer tarandus platyrhynchus*, carcases) to survive the winter (Prestrud, 1992; Samelius, 2004). Reduced predation risk may allow the ptarmigan to maximize fat reserves and locomotor efficiency, minimising starvation risk in preference to maximising escape performance. Indeed, the observed regulated reduction in fat reserves, coincident with the return of sunlight during early spring, is consistent with the notion that birds downregulate lipid storage to compensate for the increasing risk of predation at this time (Mortensen and Blix, 1985). The observation that other ptarmigan species do not fatten significantly despite similar unpredictability of resources to those on Svalbard may be a result of an increase in the likelihood of winter predation on these birds (Mortensen et al., 1983). A more detailed comparison of jumping performance in relation to predation risk and the maintenance of fat reserves in mainland rock and willow ptarmigan populations under differing levels of predation would be an interesting area for future research.

In summary, seasonal changes in the *M*_b_ of Svalbard rock ptarmigan have significant consequences for their take-off performance. A reduction in jumping ability during winter appears to be the result of an inability to increase hind-limb power output above summer levels. Although predator escape may be significantly reduced as a result, this loss of performance may be irrelevant to wild birds in light of their lower winter predation risk on Svalbard. Nonetheless, these results illustrate the importance of predation risk in contributing to the behavioural strategies of birds and the regulation of optimal lipid levels.

## MATERIALS AND METHODS

### Ethics statement

All experimental procedures were covered by a UK Home Office project licence (40/3001) held by Dr Codd and under ethical approval of the National Animal Research Authority of Norway (permit number 1333/2008) and the University of Manchester.

### Animal husbandry

The experimental group consisted of captive adult male Svalbard rock ptarmigan, housed at the Department of Arctic Biology, University of Tromsø, Norway. Experiments were conducted on the same birds during summer (*n* = 5, *M*_b_ = 551.5±9.4 g) and winter (*n* = 4, *M*_b_ = 806.6±26.3 g). All means are displayed as ± the standard error. Birds were maintained indoors with *ad libitum* access to high quality food and water in line with previous studies (Lees et al., 2013; Lees et al., 2012; Lees et al., 2010; Nudds et al., 2011). Artificial light and temperature conditions matched those in Tromsø (69°46′N), ensuring that all birds underwent their natural seasonal physiological changes (Mortensen et al., 1983; Stokkan et al., 1995). The birds were previously used in studies concerning locomotor energetics (Lees et al., 2012; Lees et al., 2010) and so had been trained to locomote on a treadmill. No jump training was necessary prior to measurements as individuals already exhibited a strong tendency to jump during both seasons.

### Experimental protocol

**Fig. 4.**
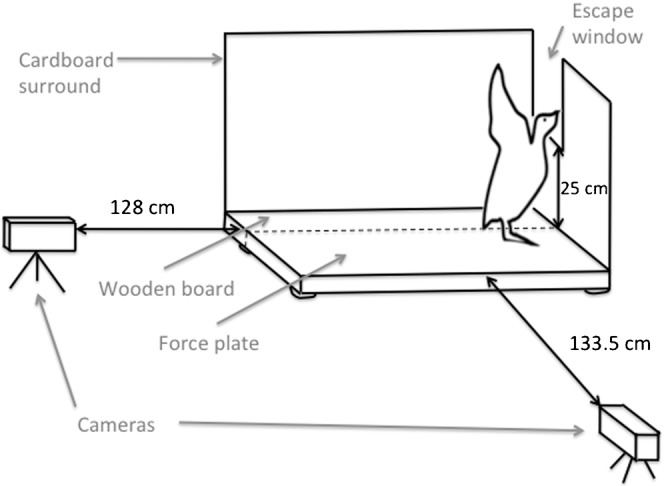
Diagram of the force-plate set-up used to determine ptarmigan jump performance.

The jumping platform consisted of a force platform (9286B, Kistler Instruments Ltd, Winterthur, Switzerland, natural frequency ≈200 Hz in the *z*-axis and 350 Hz in the *x*- and *y*-axes) positioned adjacent to a wooden board of the same height and length (Fig. 4) (with around a 1 mm gap between the two). Stiff rubber matting covered this platform so that the birds could not distinguish between the two different substrates. To ensure that birds jumped, the platform was bordered on two sides by a cardboard wall. A section of the wall in front of the bird was cut to create a 25 cm high escape window that elicited the desired jumping behaviour. The dimensions and orientation of the platform and walls were such that when placed on the platform, birds had one leg on the force plate and one on the wooden board, allowing measurement of forces from just one leg (in this case the right leg), following the method of Henry et al. (Henry et al., 2005). During trials, birds were filmed laterally at 100 Hz using a Sony Handycam HDR-XR520 (Sony, Tokyo, Japan) placed 133.5 cm from the platform. To verify that only one foot was in contact with the force plate during trials, footage was also obtained from a posterior camera at 25 Hz (Sony Handycam HDR-SR8E, Sony, Tokyo, Japan) 128 cm from the rear of the force plate. Voltage outputs from the force platform were acquired at 500 Hz using BioWare (Kistler Instruments Ltd, Winterthur, Switzerland). Zeroing the output and then loading it with a known weight calibrated the force plate. Tapping a 500 g metal weight onto the force plate at the end of each trial and matching the force peaks to the single video frames in which impact occurred was used to synchronize video and force-plate data.

Prior to trials, reflective markers were placed in line with the sternum and at the tip of the claw of the birds to allow determination of the start and end of a jump, respectively. A marker was also placed at the mid synsacrum directly above the hip joint in order to allow calculation of the take-off body angle measured as the angle of a line drawn through the synsacral and sternal markers relative to the horizontal, as in Earls (Earls, 2000). During trials, birds were placed onto the jumping platform directly beneath the escape window to ensure that birds only performed vertical jumps, allowing comparison of jumping performance in the absence of alterations to take-off angle. Birds were allowed to settle and were than startled into a jump escape response by clapping. Some birds jumped without prompting and the data from these jumps were included in the dataset as forces were the same as those in induced jumps. The mean number of trials per bird was 3.75±1.6 during winter (total of 15 jumps) and 5.2±0.6 during summer (total of 26 jumps).

### Jump performance calculations

Performance variables of horizontal (*a*_H_) and vertical (*a*_V_) acceleration were calculated from the measured horizontal (*F*_H_) and vertical (*F*_V_) forces as *a*_H_ = *F*_H_/*M*_b_ and *a*_V_ = (*F*_V_−g*M*_b_)/*M*_b_. Total forces were assumed to be double those of the measured (single leg) forces. *a*_H,fp_ and *a*_V,fp_ were integrated to calculate horizontal (*V*_H_) and vertical (*V*_V_) velocity, respectively. The starting point (time 0 for all of the time points discussed below) of the jump used for integration was determined from the video data as the instant during which the sternal marker no longer moved downwards but reversed direction and started its upwards motion (Earls, 2000). The end of the jump was defined as the point at which the toe marker lost contact with the ground. Powers were obtained by multiplying velocity and force from which the peak value across the time course of the jump was obtained. Average power was simply the average power value across the time period of the jump. Power was not integrated across time so work was not calculated.

### Statistical analyses

The mean data for each individual were used for all statistical analyses. Differences in the magnitude and occurrence of peak vertical and horizontal forces, velocities and powers were determined using independent samples *t*-tests. Similarly, differences in the occurrence times of take-off parameters including crouch duration, downstroke, toe-off and take-off body angle were established using the independent samples *t*-tests. All statistical tests were performed using SPSS (SPSS v.20; IBM, Somers, NY, USA) and means are displayed as ± standard error.

### List of abbreviations

*θ*  =  take off angle

*F*_H_  =  horizontal force

*F*_V_  =  vertical force

*M*_b_  =  body mass

*P*_H_  =  horizontal power

*P*_sum_  =  total power

P*_sum_  =  average body mass-specific total power

*P*_V_  =  vertical power

*V*_H_  =  horizontal velocity

*V*_V_  =  vertical velocity
